# Extrinsic and intrinsic control of striatal cholinergic interneuron activity

**DOI:** 10.3389/fnmol.2025.1528419

**Published:** 2025-02-13

**Authors:** Desh Deepak Ratna, Tanner Chase Francis

**Affiliations:** Department of Drug Discovery and Biomedical Sciences, College of Pharmacy, University of South Carolina, Columbia, SC, United States

**Keywords:** neuropeptides, neuromodulators, cholinergic interneurons, striatum, dopamine, acetylcholine, substance P, plasticity

## Abstract

The striatum is an integrated component of the basal ganglia responsible for associative learning and response. Besides the presence of the most abundant *γ*-aminobutyric acid (GABA-ergic) medium spiny neurons (MSNs), the striatum also contains distributed populations of cholinergic interneurons (ChIs), which bidirectionally communicate with many of these neuronal subtypes. Despite their sparse distribution, ChIs provide the largest source of acetylcholine (ACh) to striatal cells, have a prominent level of arborization and activity, and are potent modulators of striatal output and play prominent roles in plasticity underlying associative learning and reinforcement. Deviations from this tonic activity, including phasic bursts or pauses caused by region-selective excitatory input, neuromodulator, or neuropeptide release can exert strong influences on intrinsic activity and synaptic plasticity via diverse receptor signaling. Recent studies and new tools have allowed improved identification of factors driving or suppressing cholinergic activity, including peptides. This review aims to outline our current understanding of factors that control tonic and phasic ChI activity, specifically focusing on how neuromodulators and neuropeptides interact to facilitate or suppress phasic ChI responses underlying learning and plasticity.

## 1 Introduction

The striatum is the largest portion of the basal ganglia and provides information required for movement, motivation, and sensory processing. In rodents, the striatum is typically divided into the dorsal striatum (DSt) and ventral striatum composed of the nucleus accumbens (NAc) and olfactory tubercle (Chen et al., 2020). The striatum consists of primarily inhibitory GABAergic cells that receive significant excitatory, glutamatergic input from various regions including cortex, thalamus, and other limbic structures (Haber, 2011; Gerfen and Bolam, 2016; Hunnicutt et al., 2016). Additionally, they also receive local inputs releasing substance P and GABA (Blomeley and Bracci, 2008; Pickel et al., 2000; Francis et al., 2019; Tepper et al., 2004). The predominant GABAergic neurons are primary projection neurons of the striatum, known as spiny projection neurons or medium spiny neurons (MSNs), which integrate these excitatory, inhibitory, and modulatory inputs to produce movement and motivated action, and this activity is modulated by synaptic plasticity. MSNs require global and local modulatory signaling, notably dopamine (DA) and acetylcholine (ACh) signaling, to produce lasting excitatory and inhibitory plasticity to promote behavioral learning and response (Aosaki and Kawaguchi, 1996; Aosaki et al., 1998; Hunnicutt et al., 2016; Klawonn and Malenka, 2018). However, several peptides have recently been observed to strongly control local activity and response (Picciotto, 2008; Castro and Bruchas, 2019; Belilos et al., 2023). These modulatory signals from global and local sources converge to drive long-term potentiation (LTP), long-term depression (LTD) (Xu et al., 2020; Rana et al., 2022), and ensemble activity (Pennartz et al., 1994; O’Donnell, 1999), critical for striatal function and behavior.

The primary source of ACh is local cholinergic interneurons (ChIs), which make up only 1% of the total cells within the region. ChIs are widely distributed throughout the striatum and are found in low density. Despite the small density of cells, ChIs have large axonal and dendritic arbors, allowing them to influence activity by providing broad, synchronous signals to MSNs and other neuronal cell types in striatum such as neuropeptide Y (NPY)-expressing and tyrosine hydroxylase-expressing interneurons (English et al., 2012; Matamales et al., 2016; Kocaturk et al., 2022). ChI activity often anti-correlates with midbrain activity and DA release during a widely described “conditioned pause” response, which opens windows in which signaling can facilitate plasticity (Kimura et al., 1984; Aosaki et al., 1994a; Graybiel et al., 1994; Morris et al., 2004). To provide temporally locked release of ACh, this response relies on: (1) intrinsic mechanisms; (2) classical neurotransmitters that act on post-synaptic sites causing direct excitation or inhibition including glutamatergic and GABAergic neurons; and (3) neuromodulatory systems including dopamine, acetylcholine, and peptides. ChIs in both the dorsal and ventral striatum play key roles in regulating various behavioral functions, including motivation, reward, learning, feeding, pleasure, motor control, and decision-making (Haber, 2011; Warner-Schmidt et al., 2012; Atallah et al., 2014).

Novel tools such as neurotransmitter and neuromodulator sensors have provided a new understanding into the mechanisms of this phasic transmission. These studies, paired with current and previous pharmacological studies, have given deep insight into the necessity and sufficiency of ACh in controlling different forms of excitatory plasticity on MSNs and other cell types. Here, we review how ChIs are excited or inhibited to provide a basis for understanding what factors participate in phasic ChI responses underlying plasticity. In addition, we discuss a newly described mechanism for peptide control over these phasic responses and how this response facilitates plasticity in the striatum.

## 2 ChI properties

### 2.1 Morphology and distribution

ChIs are distinctive with large soma and aspiny dendrites with a high order of axonal arborizations (Pickel et al., 2000; Tepper and Bolam, 2004). These neurons can be distinguished by choline acetyltransferase (ChAT) and vesicular ACh transporter (VAChT) expression. ChIs can self-regulate local ACh through their expression of acetylcholinesterase (Gonzales and Smith, 2015). While the developmental origin of NAc and DSt ChIs arise from the septal epithelium during development, the DSt also contains ChIs arising from the medial ganglionic eminence and preoptic area, which may drive diversity in ChI function and activity (Allaway and Machold, 2017; Ahmed et al., 2019). In rodents, ChIs are higher in density in dorsolateral regions in the rostral portions of the striatum and tend to be higher in density in rostral regions, compared to more caudal regions (Meredith et al., 1989; Matamales et al., 2016; Gonzales and Smith, 2015).

### 2.2 Intrinsic control of ChI activity

**Figure 1:**
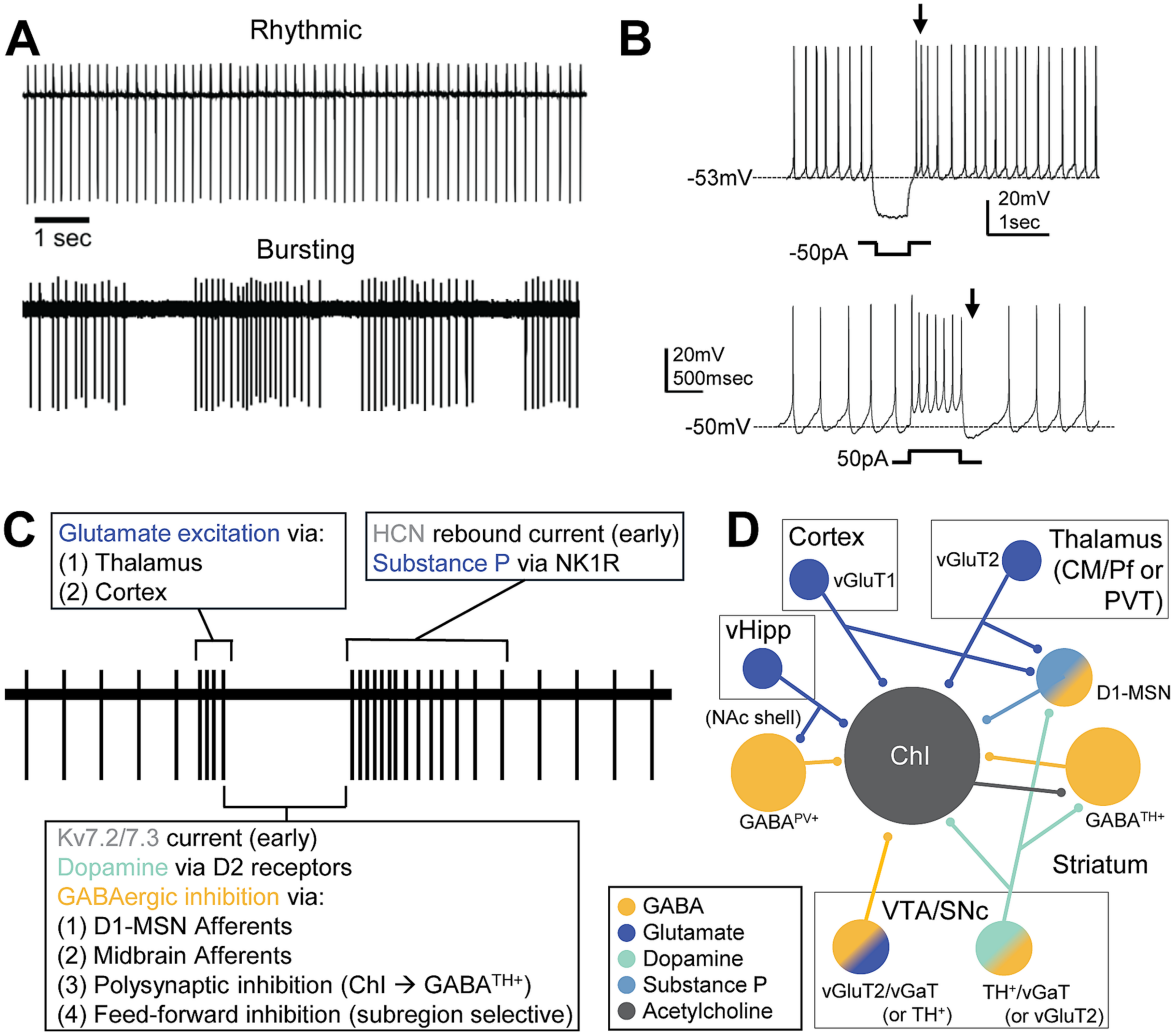
Firing pattern mechanisms of ChIs. **(A)** *Ex vivo* cell attached recordings showing rhythmic and bursting activity of ChIs. **(B)** Whole cell patch clamp of ChIs *ex vivo* shows rebound firing caused by *I_h_* current after current injected to inhibit the ChI (top) and, after current is injected to excite the ChI, inhibition caused by an after hyperpolarization initiated by Cav1 channels and a Kir current followed by depolarization by HCN and persistent sodium currents (bottom). **(C)** Phases of the conditioned pause response and known mechanisms driving each phase. Glutamatergic excitation causes bursting activity followed by DA and GABA mediated pause or decrease in firing. Rebound firing occurs followed by extended excitation by the neuropeptide substance P. **(D)** Local and afferent inputs to ChIs that have been identified to be critical in the conditioned burst-pause-rebound response. Cortical and thalamic inputs activate both ChIs and MSNs via glutamate (dark blue). D1-MSNs activated by this input are partially responsible for the initial GABAergic inhibition (yellow) followed by the release of substance P (light blue). GABA interneurons, ChIs, and D1-MSNs receive midbrain dopamine (teal) which modulates ChI activity. vHipp inputs to GABA^PV+^ neurons promote feedforward inhibition to ChIs in the NAc shell. ChIs receive excitatory glutamatergic and inhibitory GABAergic input from midbrain VTA neurons. These neurons could be glutamate only, GABA only, DA only, or a mix which are intermixed and differentially distributed across midbrain sub-compartments. Abbreviations: centromedian/parafascicular nucleus of the thalamus (CM/Pf), cholinergic interneuron (ChI), dopamine 2 receptor (D2R), nucleus accumbens (NAc), neurokinin 1 receptor (NK1R), paraventricular thalamus (PVT), substantia nigra pars compacta (SNc), tyrosine hydroxylase (TH), ventral hippocampus (vHipp), ventral tegmental area (VTA), vesicular GABA transporter (vGAT), vesicular glutamate transporter (vGluT).

ChIs are tonically active neurons spontaneously firing around 5–10 Hz and these tonic activity patterns are autonomous, without the need for any input (Wilson et al., 1990). ChI activity primarily releases ACh but a subset of ChIs have been documented to also express vGlut3 and directly release glutamate or facilitate glutamate release via presynaptic nicotinic receptors (Higley et al., 2011). ChIs display several distinct patterns both *ex vivo* and *in vivo*: bursting, irregular, and rhythmic firing or “pacemaking” firing (Goldberg and Wilson, 2005; Sharott et al., 2012; Shroff et al., 2023) (Figure 1A). In addition, mixed mode ChIs are observed in the NAc shell that differs across sex (Olson et al., 2024). The mode of these cells can change with the blockade of Cav2.2 calcium channels which activates a SK potassium channel current, causing the mode to change from rhythmic to bursting (Bennett et al., 2000; Goldberg and Wilson, 2005; Goldberg and Wilson, 2016). The tonic activity of ChIs relies on calcium channels, particularly Cav2.1 and 2.2, and several potassium conductances, including the BK and SK potassium currents which mediate the repolarization and medium afterhyperpolarization potential, respectively. Cav1 causes a slower afterhyperpolarization potential and inward rectifying potassium currents can prolong the hyperpolarization (Goldberg et al., 2009). ChIs experience a large voltage sag during inhibition, characteristic of the hyperpolarization-activated cyclic nucleotide-gated (HCN) channel-mediated I_h_ potassium current (Oswald et al., 2009), which drives rebound spiking (Figure 1B). In addition, the Kv7.2/7.3 mediated delayed rectifier I_Kr_ potassium current (Zhang et al., 2018) or Kv1 (Tubert et al., 2024) drives inhibition after burst firing (Figure 1B). This inhibition provides the ability to hyperpolarize and suppress ChI firing and may be a significant part of the inhibitory current in phasic pause responses (Zhang et al., 2018).

## 3 Neurotransmitter and neuromodulator contributions to ChI phasic activity

Despite the intrinsic firing patterns of the ChIs, synaptic input can play a prominent role in altering activity patterns and synchronization. *In vivo*, ChIs synchronize their firing in response to behaviorally relevant stimuli (Ravel et al., 1999; Nougaret and Ravel, 2015) and undergo a pause burst pattern termed the “conditioned pause response” (Schulz and Reynolds, 2013; Zhang and Cragg, 2017). This response is thought to be driven by many diverse afferent and local sources, as listed in Table 1.

**Table 1:** Changes in striatal cholinergic interneuron activity by various neurotransmitters and neuromodulators.

Neurotransmitter/modulator	Receptor (S)	Treatment	Effect on ChI activity	References
Acetylcholine	mAChR (M2)	Muscarine/oxotremorine	Decreased activity (hyperpolarization)	Calabresi et al. (1998a,b)
	mAChR (M2)	AF-DX 116 (antagonist)	Increased ACh release (*in vivo* microdialysis)	Galarraga et al. (1999)
	nAChR	Nicotine	Increased ACh release (radioassay)	Sandor et al. (1991)
	mAChR	Muscarine	Decreased activity	Yorgason et al. (2017)
Corticotropin-releasing factor (CRF)	CRF1	CRF	Increased activity (increased activity)	Lemos et al. (2019)
	CRF	CRF	Increased release of ACh in NAcS (microdialysis)	Chen et al. (2012)
Dopamine	D1	DA	Increased activity	Aosaki et al. (1998)
	D1	Cocaine	Increase ACh release (*in vivo* microdialysis)	Consolo et al. (1996), Berlanga et al. (2003)
	D2	Quinpirole	Decreased activity (reduction of N-type Ca^2+^ currents)	Yan et al. (1997)
	D5	Dopamine	Decreased activity (enhanced Zn2+ sensitive component of GABA_A_ currents)	Yan et al. (1997)
Dynorphin	MOR	DAMGO	Decreased firing	Ponterio et al. (2013)
GABA	GABA_A_	Picrotoxin	Decreased EPSP	Pisani et al. (2002)
	GABA_A_	Muscimol (agonist)	Decrease ACh release (*in vivo* microdialysis); all effects were seen at high but not low drug infusion concentration	DeBoer and Westerink (1994)
	GABA_A_	Bicuculline (antagonist)	Increase in ACh release (*in vivo* microdialysis)	
	GABA_B_	Baclofen (agonist)	Decrease ACh output (*in vivo* microdialysis)	
	GABA_A_	nAChR (antagonist)	Block IPSC generated by electrical stimulation	Sullivan et al. (2008)
	GABA_A_	Diazepam	Increased GABA_A_ current in ChAT cell (inhibition of chat cell)	Yan and Surmeier (1997)
Galanin	GALR1	Galanin	Increased ACh release (microdialysis)	Ogren and Pramanik (1991), Pramanik and Ogren (1993)
Glutamate	NMDA	MK801 (antagonist)	Reduced activity (reduce amplitude, and duration of EPSP)	Pisani et al. (2002)
	AMPA	CNQX (AMPA antagonist)	Reduced activity (reduce amplitude of EPSP)	
	NMDA	CIQ (allosteric modulator)	Increase activity (increased firing rate)	Feng et al. (2014)
	mGluR1/5	DHPG (agonist)	Increased activity (depolarized)	Pisani et al. (2002), Bell et al. (2002)
	mGluR1 and 5	DHPG (agonist)	Increased activity (depolarized)	Bonsi et al. (2005)
	NMDA	MK-801	Decreased ACh release (*in vivo* microdialysis)	Consolo et al. (1996), Baldi et al. (1995)
Insulin	InsR	HNMPA blocked increased activity after 3 s stimulation	Increased excitability	Stouffer et al. (2015)
Leptin	LepRb	Leptin	Increased excitability	Mancini et al. (2022)
Opioid	mu	DAMGO	Decreased activity (inhibits firing rate)	Ponterio et al. (2013), Arttamangkul et al. (2021)
	mu	Endomorphine	Decreased activity (inhibits firing rate)	Britt and McGehee (2008)
	mu/delta	Met-enkephalin (ME) (agonist)	Decreased (inhibit firing)	Yorgason et al. (2017)
	Delta	Deltorpin (agonist)	Decreased activity (inhibits firing rate)	Arttamangkul et al. (2021)
Serotonin	5-HT2	5-HT (agonist)	Increased activity (increased firing)	Blomeley and Bracci (2005)
	5-HT1	5-HT	Decreased in ACh release (microdialysis)	Rada et al. (1993)
	5HT1	5-HT	Increased activity (depolarization in dorsal striatum)	Virk et al. (2016)
	5-HT1B	5-HT	Reduced activity (hyperpolarization in ventral striatum)	Virk et al. (2016)
Substance P (SP)	NK1	SP	Increased activity (increased firing)	Francis et al. (2019)
	NK1	SP	Increased activity (depolarization)	Aosaki and Kawaguchi (1996)
	NK1, NK2, NK3	SP, Neurokinin A4-10, Senktide (agonist)	Increased activity (depolarization)	Preston et al. (2000)

Salient sensory stimuli, rewarding stimuli, and aversive stimuli during classical conditioning and instrumental tasks produce a stereotyped, multi-phasic, pause-rebound response in ChIs (Figure 1C) (Apicella et al., 1997; Morris et al., 2004; Schulz and Reynolds, 2013; Nougaret and Ravel, 2015; Zhang and Cragg, 2017). While salient stimuli alone can cause some ChIs to elicit this response, more ChIs are recruited after conditioning this stimulus to a rewarding or aversive outcome (Aosaki et al., 1994b; Nougaret and Ravel, 2015). Additionally, this response occurs in some ChIs, but not all, and may represent distinct sensory stimuli (Aosaki et al., 1994b; Apicella et al., 1997). However, the features of these responses appear consistent across these stimuli and are conserved across mammals. These responses have been observed in rodents as well as primates when assessing *in vivo* firing rates (Apicella et al., 1997; Ravel et al., 1999), calcium fluorescence signals from ChIs (Hegedüs et al., 2023), and ACh release via ACh sensor fluorescence (Gallo et al., 2022; Belilos et al., 2023). These conserved responses are significant because they underlie neuronal plasticity, which is important for learning, sensory discrimination, and motivated response (Zhang and Cragg, 2017). In addition, this pause-rebound is observed across all subregions of the striatum, with varying strength and duration of each signal (Duhne et al., 2024). The overarching mechanisms of this response have been debated but appear to involve multiple mechanisms, including several excitatory inputs to ChIs, intrinsic ChI electrophysiological properties, neuromodulators including DA (Paladini and Roeper, 2014; Goldberg and Wilson, 2016; Zhang and Cragg, 2017), and likely require other synaptic signaling factors including some peptides. While there are various ChI activity responses to sensory stimuli (Zhang and Cragg, 2017), here we discuss the classical burst-pause-rebound response and distinguish between factors that drive each phase. The mechanisms contributing to each behavior and subregion are summarized in Figures 1C,D and discussed in detail below.

### 3.1 Burst excitation via glutamate

ChIs are tonically active and continue to fire action potentials in the absence of any input (Bennett and Wilson, 1999). Glutamate receptor expression plays a significant role in promoting time-locked ChI activity and synchrony, influencing plasticity on local neurons and MSNs. Fast neurotransmission through AMPA receptors drives bursting activity in more tonic-firing populations (Colquhoun et al., 1992; Ding et al., 2010). All AMPA receptor subunits are expressed on ChIs (Calabresi et al., 1998a; Bell et al., 2002; Pisani et al., 2002; Deng et al., 2007) with subunits GluR_1_, GluR_3_, and GluR_4_ contributing to a high calcium permeability (Götz et al., 1997). ACh release is critically dependent on NMDA receptor activation on ChIs (de Jesus Aceves Buendia et al., 2019). *In situ* hybridization studies with double labeling reveal mRNA expression for NMDAR_2D_ and lower levels of NMDAR_2B_ and NMDAR_1_ subunits in ChIs (Landwehrmeyer et al., 1995). Additionally, single-cell PCR analysis and double-label *in situ* hybridization have detected low expression of NMDAR_2A_ subunits (Standaert et al., 1999; Richardson et al., 2000). Allosteric modulation of NMDARs by bath application with CIQ (positive allosteric modulator for NMDA) enhances the spontaneous firing rate of ChIs (Feng et al., 2014).

Glutamatergic inputs to ChIs arise predominantly from the cortex and the thalamic parafascicular nucleus (PF) (Kemp and Powell, 1970; Scatton and Lehmann, 1982; McGeorge and Faull, 1989; Lapper and Bolam, 1992; Groenewegen and Berendse, 1994; Thomas et al., 2000; Ding et al., 2010, 2014; Smith et al., 2014; Guo et al., 2015; Klug et al., 2018; Assous and Tepper, 2019; Mandelbaum et al., 2019). These glutamatergic terminals can be differentiated by their origin: cortical terminals express vGluT1, while thalamic terminals express vGluT2 (Fremeau et al., 2004). In rats, the intralaminar thalamic nuclei serve as the primary origin of thalamostriatal projections (Berendse and Groenewegen, 1990; Erro et al., 2001; Van Der Werf et al., 2002) with substantial projections arising from centromedian-parafascicular (CM-PF) nuclei. Although both these nuclei send projections to ChIs, neuronal tracing in transgenic mice has shown that ChIs receive more projections from cortical neurons compared to the thalamus (Guo et al., 2015), but with less synaptic contact (Sadikot et al., 1992; Doig et al., 2014). Also, there is a difference in the synaptic localization onto the ChIs. Distal dendrites of ChIs receive synaptic input from cortical terminals. In contrast, soma and the dendritic shaft receive synaptic connections from thalamic terminals (Lapper and Bolam, 1992; Dimova et al., 1993; Sidibé and Smith, 1999; Thomas et al., 2000). Single-pulse electrical stimulation of cortical and thalamic neurons elicits different responses in ChIs. Cortical stimulation results in strong spiking activity, whereas thalamic stimulation induces weaker spiking. However, during trains of high-frequency stimulation, the probability of ChIs firing gradually decreases for cortical inputs but increases for thalamic inputs (Ding et al., 2014). This region-driven change in firing is driven by synaptic facilitation from thalamic inputs and accommodation from cortical inputs (Mamaligas et al., 2019).

Cortical and thalamic input can drive the initial excitation and may be responsible for the initial inhibitory pause via nicotinic DA release (Ding et al., 2010; Oldenburg and Ding, 2011). Cortical inputs are associated with the strongest initial ChI activity through direct excitation. Cortical stimulation can produce the initial excitation, pause, and rebound responses and facilitate DA release following excitation (Kosillo et al., 2016). Intralaminar thalamic inputs are implicated in the initial burst response through direct depolarization and activation of ChIs (Ding et al., 2010; English et al., 2012; Doig et al., 2014), and activity of these inputs are sustained through the initial phase of the pause. In particular, the CM/Pf region of the thalamus in primates and lateral and medial parafascicular thalamic nuclei are necessary for all three phases (Matsumoto et al., 2001; Yamanaka et al., 2018). Pauses and rebounds are attenuated after muscimol-mediated neuronal inactivation of these thalamic regions (Matsumoto et al., 2001). Despite this, the pause can occur in the absence of this initial excitation, indicating that multiple mechanisms may cause this excitation.

In the striatum, DA terminals also exhibit neurotransmitter co-release, including glutamate. In the medial ventral tegmental area (VTA) there are a variety of cell types with differential neurotransmitter release: glutamate only, glutamate with GABA, and GABA only (Miranda-Barrientos et al., 2021). DA neurons from this region tend to project to medial striatal subregions (Tecuapetla et al., 2010; Poulin et al., 2018; Chuhma et al., 2023; Voorn et al., 2004). The DA neurons in the lateral VTA are reported to co-release GABA with DA or DA alone (Kawano et al., 2006; Tritsch et al., 2012; Poulin et al., 2018; Steinkellner et al., 2018; Mingote et al., 2019). As evidence of this spatial segregation, midbrain glutamate inputs can excite ChIs in the striatum where the strongest excitation seen is in the medial shell region of the NAc (Chuhma et al., 2023). Glutamatergic input from VGluT2 expressing DA neurons in the medial portions of the VTA can produce burst excitation followed by a pause as well in the medial shell of the NAc (Chuhma et al., 2014). Some portions of the DSt defies this topology and it has been shown stimulation of midbrain inputs to this region excite ChIs (Straub et al., 2014; Mingote et al., 2019). This is driven by activation of metabotropic glutamate receptors (mGluRs) or D5 receptors which excite ChIs in the lateral DSt via TrpC3/7 channels (Chuhma et al., 2018). Overall, glutamate from multiple sources may contribute to these initial burst responses but is spatially differentiated by striatal sub-compartments.

### 3.2 Factors contributing to the pause

The mechanisms of the pause phase in ChI activity have been described and studied most prominently (Cragg, 2006; Brown et al., 2012; Zhang and Cragg, 2017; Zucca et al., 2018). This pause is a period of decreased or no ChI activity that is synchronous spatially across ChIs. This phenomenon requires several identified mechanisms, including DA inhibition, delayed rectification by intrinsic currents, and GABAergic inhibition (Sullivan et al., 2008; Aosaki et al., 2010; Brown et al., 2012; Tubert et al., 2024).

#### 3.2.1 Dopamine

The most studied neuromodulator released in the striatum is DA. Dopaminergic inputs to the striatum primarily originate from the mesolimbic and nigrostriatal systems (Björklund and Dunnett, 2007; Nieh et al., 2013). These DA afferents are topographically arranged within the striatum. Projections from the VTA to NAc follow a medial-to-lateral topography where the medial shell of the NAc receives inputs from the posterior-medial VTA, while lateral shell and core receive inputs from the lateral VTA, with no overlap in the projecting VTA-DA neurons (Swanson, 1982; Beier et al., 2015; Luo et al., 2018; Breton et al., 2019; Mingote et al., 2019; Beier, 2022). The DA projections to the DSt, originate from the most lateral substantia nigra pars compacta (SNc) (Haber, 2011). DA signaling in the striatum occurs via both slow, long-lasting volume transmission and phasic release through discrete synaptic connections (Kubota et al., 1987; Hattori et al., 1991; Fuxe et al., 2010; Sulzer et al., 2016; Uchigashima et al., 2016; Liu and Kaeser, 2019).

Dopaminergic innervation of the striatum is well known to modulate both MSN and ChI activity (Bonsi et al., 2011; Lim et al., 2014; Mamaligas et al., 2019). ChIs are reported to co-express D2 and D5 receptors (Yan and Surmeier, 1996, 1997; Maurice et al., 2004; Castello et al., 2020). *In situ* hybridization and immunohistochemistry studies have shown co-localization of D2 receptors in ChAT-positive ChIs (Le Moine et al., 1990; Khan et al., 2000). D5 receptors are expressed in all striatal ChIs at levels significantly greater than those found in other striatal neurons. Electron microscopy and immunostaining studies further show that D5 receptors are localized to ChIs (Bergson et al., 1995; Calabresi et al., 2000; Khan et al., 2000; Rivera et al., 2002), where they are predominantly expressed in cell bodies and on primary dendrites (Centonze et al., 2003). In slice preparations completely lacking the D1 receptor, DA application depolarized ChIs suggesting that D5 receptors are responsible for increasing ChI activity (Centonze et al., 2003). This depolarization is through mGluR and D5 receptor-mediated activation of TrpC3/7 channels in the lateral DSt, and mGluR only in the medial DSt (Chuhma et al., 2018). This depolarization was also evident following D1/D5 receptor agonism which significantly suppressed the pause response independently of HCN channels (Tubert et al., 2024). In a DA-depleted state, inverse agonism of the D5 receptor restores the pause response. Overall, this suggests activity of the D5 receptor plays a prominent role in inhibiting the pause response in both DA-depleted and DA-intact conditions.

The inhibitory D2 receptor is more prominently expressed than the D5 receptor, leading to the predominantly inhibitory effects of DA on ChIs (Yan et al., 1997). In response to salient stimuli, a pause in the tonic activity of ChIs is observed, triggered by a burst of midbrain DA in response to reward-associated or aversion-associated cues and events (Schulz and Reynolds, 2013; Straub et al., 2014; Kim et al., 2019) via inhibitory D2 receptors (Ikarashi et al., 1997; Watanabe and Kimura, 1998). This aligns with findings that show D2 agonists reduce the firing frequency of ChIs (Maurice et al., 2004; Wang et al., 2013) and DA application significantly diminishes spontaneous firing rates of ChI neurons *in vivo* (Rolls et al., 1984). The pause in ACh release can be completely blocked *in vivo* by the D2 receptor antagonist sulpiride in the DSt (Watanabe and Kimura, 1998), and NAc (Gallo et al., 2022), and D2 receptor overexpression can enhance this response (Gallo et al., 2022). This outcome is also evident *ex vivo* with electrically stimulated pause responses being diminished by decreased DA expression via a 6-OHDA lesion (Sanchez et al., 2011), suppressing D2 signaling pharmacologically (Ding et al., 2010), or knocking out D2 receptors (Kharkwal et al., 2016). *Ex vivo* nigrostriatal afferents to DSt ChIs produce a D2 receptor-sensitive pause, and a single action potential elicited from SNc activation causes all described phases (Straub et al., 2014). In addition, blocking D2 receptor activation can allow for excitation. Suppression of D2 receptor activation with sulpiride promotes burst firing followed by a brief decrease in firing in most ChIs during phasic stimulation of SNc afferents via unmasking of mGluR signaling (Cai and Ford, 2018). Overall, this suggests that D2 receptor signaling is critically important for the pause response.

#### 3.2.2 Acetylcholine

ChIs are the primary source of ACh in the striatum. ACh can directly control ChI activity and indirectly control signaling contributing to the pause response (Goldberg and Wilson, 2016). ACh released in the striatum, whether from local or external sources, acts through muscarinic ACh receptors (mAChRs) and nicotinic ACh receptors (nAChRs) (Oldenburg and Ding, 2011). In the striatum, mAChRs are present on ChIs (Weiner et al., 1990; Ding et al., 2006; Oldenburg and Ding, 2011), with M4 subtype being the most abundant, followed by M1 and M2 subtypes (Yasuda et al., 1993; Chapman et al., 2011). M2 and M4 AChRs are coupled to Gi/o inhibitory signaling and suppress voltage-activated calcium channel (Cav2) currents while enhancing inward rectifying potassium channel (Kir3) currents (Caulfield and Birdsall, 1998; Brown, 2018). M2 and M4 AChRs are expressed presynaptically on ChIs and function as autoreceptors to reduce ACh release (Hersch et al., 1994; Ding et al., 2006; Ztaou et al., 2016). Blocking M2 receptors increases ACh release in the striatum (Lachowicz et al., 2001) and muscarinic agonists inhibit ACh release not only in the striatal slices but also in hippocampal and cortical slice preparation (Zhang et al., 2002). In contrast, the genetic knockout mouse model reveals that autoinhibition of ACh release in striatum is mainly regulated by M4 receptor (Zhang et al., 2002).

nAChRs are expressed post-synaptically on ChIs or pre-synaptically on afferent terminals and are composed of various subunits that can form either homomeric or heteromeric complexes (Albuquerque et al., 2009; Dani, 2015; Ho et al., 2020). Alpha (α2-10) and beta (β2-4) are the most prominently expressed nAChR subtypes in the striatum (Zoli et al., 2002; Exley and Cragg, 2008; Gotti et al., 2009; Feduccia et al., 2012). Hybridization studies have shown that ChIs express α7- and β2- containing nAChRs, which play key roles in behaviors related to anxiety, social interactions, and exploration (Abbondanza et al., 2022). Deleting these receptors significantly alters several behavioral parameters, particularly as mentioned above (Abbondanza et al., 2022).

ACh and DA release are concurrently released and often covary. That is, when DA is released, ChI activity is decreased, resulting in low ACh release (Barbeau, 1962; Morris et al., 2004; Brown et al., 2012; Chuhma et al., 2014; Straub et al., 2014; Chantranupong et al., 2023). The temporal coordination of ACh and DA dynamics in striatum reflects the intrinsic rhythms of these neurotransmitters resulting in locally synchronized activity of the ChIs (Krok et al., 2023). Interaction between ACh and DA has been observed to play a key role in facilitating the pause response. The pause response is dependent on DA release, and nAChR activation can promote its release from DA terminals. Local ACh released modulates DA signaling through nAChRs and mAChRs located on DA axons in the striatum. Notably, DA release increases following selective and synchronous activation of ChIs via nAChRs (β2-containing) (Giorguieff et al., 1976; Clarke and Pert, 1985; Kulak et al., 1997; Wonnacott et al., 2000; Cachope et al., 2012; Exley et al., 2012; Threlfell et al., 2012; Koranda et al., 2014). In fact, activating cortical and thalamic inputs to the striatum promotes ChI activity, and increased ACh release can lead to more DA release (Threlfell et al., 2012; Kosillo et al., 2016). Glutamate released by ChIs may also play a secondary role in addition to the ACh-mediated presynaptic DA release in striatum (Wonnacott et al., 2000; Kljakic et al., 2017). AMPA receptors antagonist 2,3-dihydroxy-6-nitro-7-sulfamyl-benzo (f) quinoxaline (NBQX) has been shown to attenuate the amplitude of excitatory postsynaptic potentials in MSNs (Cachope et al., 2012), suggesting a multi-synaptic effect is responsible for ACh-mediated DA release. Interestingly, ACh release spatially scales DA release through ACh waves acting on nicotinic receptors (Matityahu et al., 2023). In the medial NAc, ChIs increase firing following photo-stimulation of DA terminals, whereas inhibitory effects are observed throughout most other striatal regions (Chuhma et al., 2023). These inhibitory effects on ChIs are mediated via a polysynaptic pathway involving inhibitory interneurons (Dorst et al., 2020). These connections are robust, as a single action potential can suppress the tonic activity of neighboring ChIs (Dorst et al., 2020). This inhibition is discussed further in section 3.2.2.

The striatum also receives cholinergic inputs from the pedunculopontine tegmentum nucleus (PPT) and laterodorsal tegmental nucleus (LDT), as confirmed by lesion studies (McGeer et al., 1971). Immunofluorescence labeling shows cholinergic innervation of both the striatum and NAc from PPN and LDT. The dorsolateral striatum receives dense innervation from rostral PPN, whereas the caudal PPN innervates small, sparsely distributed regions in the DSt (Dautan et al., 2014). ACh projections from PPN are predominantly restricted to the anterior striatum (Dautan et al., 2014), which also receives innervation from prefrontal cortical areas (Hunnicutt et al., 2016). However, retrograde tracing studies have revealed that a considerable proportion of these projections are non-cholinergic (Dautan et al., 2014; Klug et al., 2018) and are likely glutamatergic, synapsing more selectively on ChAT+ interneurons (Klug et al., 2018). This ACh source does not enhance DA release via nAChR activation (Brimblecombe and Cragg, 2015), a property attributed to ChI ACh release. The role of these inputs on the conditioned pause response is not known.

#### 3.2.3 GABA

GABAergic input to ChIs is primarily local, driven by tonic GABAergic activity and local feedforward inhibition. GABA_A_ receptors mediate inhibitory signaling from striatal GABAergic interneurons to ChIs (Gonzales et al., 2013; Sato et al., 2014). ChIs have been reported to co-express several GABA_A_ receptor subunits, α3-4, β1-3, and γ1-3 subunits, as shown by RT-PCR analysis from individual ChIs (Persohn et al., 1992; Lim et al., 2014; Boccalaro et al., 2019).

ChIs receive extensive GABAergic inputs from MSN collaterals and GABAergic interneurons (Gonzales et al., 2013). Substance P expressing MSNs, also identified by DA 1 (D1) receptor expression, primarily synapse on ChIs. Enkephalin-positive terminals form few synapses on ChAT-positive neurons in rats, whereas substance P-positive terminals form symmetric synapses on cell bodies and proximal dendrites (Bolam et al., 1986; Martone et al., 1992; Kuramoto et al., 2007). This synaptic input from putative MSNs is different in primates. Using ultrastructural analysis with immunogold and localization along with peroxidase immunostaining, it has been shown that ChIs in primates receive GABAergic inputs, intra-striatal in origin, which are terminals from the direct (D1-MSNs) and indirect pathway (dopamine 2 (D2) expressing MSNs) (Gonzales et al., 2013). Substance P/D1-MSNs generate large inhibitory currents on ChIs (Francis et al., 2019) via GABA_A_ and GABA_B_ receptors (Martone et al., 1992; Kuramoto et al., 2007). Pauses mediated by D1-MSN activation and collateral inhibition produce a pause followed by long-lasting increases in activity that are dependent on substance P, neurokinin 1 receptor signaling (Belilos et al., 2023). It is possible that dopaminergic input to D1-MSNs and excitation via concomitant glutamatergic input and D1 receptor activation could enhance D1-MSN activation and inhibition of ChIs. However, blocking D1 receptor signaling in the DSt of primates was only able to suppress the pause response caused by a conditioned cue in a small subset of ChIs (Watanabe and Kimura, 1998), suggesting that this effect could be either selective to subtypes of ChIs or be region selective.

Local GABAergic inhibition is also sensitive to D2 receptor activity. ChIs participate in a large recurrent inhibitory network (Sullivan et al., 2008). Feedforward inhibition via polysynaptic connections from ChIs to tyrosine-hydroxylase positive (TH+) GABAergic interneurons (GABA^TH+^) can cause inhibition between local ChIs (Sullivan et al., 2008; Dorst et al., 2020). This could facilitate spatially localized inhibition of ChIs following ChI bursting activity by glutamatergic input. Increased DA can suppress this inhibition through D2 receptors, which may suggest that DA levels tightly and spatiotemporally control somatic ChI inhibition and polysynaptic inhibition.

Feedforward inhibition through other GABAergic interneurons has been hypothesized to play a role in the pause response. Local GABAergic inhibition via electrical stimulation can shunt action potential firing in ChIs (Bennett and Wilson, 1998). In monkeys, CM thalamic neuron stimulation promotes GABA_A_ receptor release of ACh, but through an unknown GABA source (Nanda et al., 2009). In the DSt, parvalbumin (PV+) interneurons and somatostatin (SOM+) have weak or no GABA_A_ receptor-mediated connectivity to ChIs (Szydlowski et al., 2013), indicating these local interneuron populations likely do not participate in feed-forward inhibition or contribute to the ChI pause in this striatal sub-compartment. However, in the NAc shell, ventral hippocampal excitatory input to PV+ interneurons can promote strong inhibition of ChIs, while the paraventricular thalamus mainly excites ChIs (Baimel et al., 2022). Overall, the effect on the pause response is likely limited as the response is rapid and shows little to no modulation by dopamine, except in the case of polysynaptic inhibition by GABA_TH_^+^ neurons.

The pause is likely partially driven by midbrain GABAergic inhibition of ChIs. Midbrain neurons can vary in their expression and co-expression. These cells can be DA-only, DA-GABAergic, GABAergic-only, and glutamatergic-GABAergic (Miranda-Barrientos et al., 2021; Chuhma et al., 2023). GABA is released from the midbrain across all striatal sub-compartments (Chuhma et al., 2023; Kim et al., 2023). In the NAc this response is varied. Optogenetic stimulation of midbrain GABAergic terminals induces inhibitory postsynaptic current (IPSC) in ChIs, effectively silencing tonic activity within the ventral NAc-shell, but not the dorsal NAc-shell (Brown et al., 2012; Al-Hasani et al., 2021). This pause in ChIs activity resembles the conditioned pause response and is reversed with a GABA_A_ receptor antagonist (Brown et al., 2012; Dorst et al., 2020). In the DSt, GABA can also be loaded and released from DA neuron terminals (Tritsch and Sabatini, 2012; Nelson et al., 2014; Stensrud et al., 2014; Kim et al., 2023) or released from midbrain inputs. Midbrain input to ChIs is strong and prevalent (Chuhma et al., 2023). ChIs are excited by co-releasing midbrain inputs that release GABA and glutamate; however, the strength of the inhibition tends to dominate the response. Therefore, GABAergic input from the midbrain may contribute to this inhibition. This has been observed *in vivo* as well. In one study, stimulation of GABAergic midbrain neurons targets and directly inhibits ChIs in a pause-like response, facilitating stimulus-outcome learning (Brown et al., 2012). These studies would suggest that direct GABAergic inhibition plays a role in the pause response.

#### 3.2.4 Intrinsic inhibition

This DA-mediated inhibition occurs in coordination with intrinsic ChI properties, particularly the slow inactivating I_Kr_ potassium currents, which are induced with the withdrawal of excitation (Zhang et al., 2018) and regulate thalamic-mediated pauses (McGuirt et al., 2022). However, this may only occur in the case of pauses that follow bursts and may only account for the initial period of inhibition (Oswald et al., 2009). In addition, the afterhyperpolarization potential could be prolonged by inhibition of the I_h_ current and sodium channels (Aosaki et al., 2010; Ding et al., 2010).

### 3.3 Rebound from pause

Rebound ChI spiking that occurs following the pause has received considerably less attention. The rebound can last up to seconds and also appears to rely on multiple mechanisms (Schulz and Reynolds, 2013). This response is not a consistently observed response across all behaviors and regions, though it is observed with most thalamic-induced pauses (Matsumoto et al., 2001; Yamanaka et al., 2018). While both appetitive and aversive conditioned stimuli produce the rebound response, aversive stimuli produce large rebound responses (Nougaret and Ravel, 2015).

#### 3.3.1 Intrinsic properties

The initial cause of the rebound is likely due to intrinsic ChI mechanisms of inhibition-induced rebound (Figure 1B) driven by HCN channels that produce the I_h_ current (Zhang et al., 2018), among other currents. However, this does not describe the extensive duration of the response in some preparations, as has been shown in the DSt (Straub et al., 2014). Nigrostriatal afferents to the DSt can produce pause and more lasting burst response (rebound) responses, where the rebound response mechanism was not identified (Straub et al., 2014). Blockade of calcium channels by cadmium and action potentials by tetrodotoxin eliminated this response, indicating that the synaptic release of another substance is causing this excitation. Aversive stimuli tend to produce the largest and longest-lasting rebound responses, which have been observed to last up to seconds (Ravel et al., 2003; Belilos et al., 2023). Therefore, this lasting excitation would require an additional source.

#### 3.3.2 Substance P

The peptide substance P has emerged as a potential candidate for the long-lasting excitation following the pause. Substance P is expressed at high levels within the striatum and is released in response to salient stimuli (Commons, 2010; Vanderah and Sandweiss, 2015; Belilos et al., 2023). Substance P modulates DA release (Brimblecombe and Cragg, 2015), its effect varies among ChI-dense regions around striosomes in the DSt (Brimblecombe and Cragg, 2015), and causes significant excitation of ChIs (Aosaki and Kawaguchi, 1996; Govindaiah et al., 2010; Francis et al., 2019) and increases ACh release (Guevara Guzman et al., 1993; Francis et al., 2019; Belilos et al., 2023). Of the three neurokinin (NK) receptors, the NK1 receptor has the highest affinity to substance P among other variants (Pennefather et al., 2004). The NK1 receptor is a Gq coupled, G-protein receptor expressed in ChIs (Bolam et al., 1986; Aubry et al., 1993; Richardson et al., 2000; Pérez et al., 2007) and localized to somatostatin interneurons and ChI dendrites and somas (Martone et al., 1992; Pickel et al., 2000; Francis et al., 2019). Substance P causes a change in ChI activity in several ways via direct and indirect effects. Direct binding to the NK1 receptor results in persistent yet unidentified sodium current. When substance P is in high concentration *ex vivo* (250 nM – 1 μM), it can lead to long-lasting excitation lasting for minutes (Francis et al., 2019). However, shorter excitation due to substance P has been observed *in vivo* at physiological concentrations (Belilos et al., 2023). Indirectly, substance P can also suppress GABA_A_ receptor-mediated IPSCs, resulting in increased ACh release from ChIs (Arenas et al., 1991; Govindaiah et al., 2010). This resulting effect of increased ACh release through direct or indirect mechanisms has been shown to have excitatory (Pakhotin and Bracci, 2007; Belilos et al., 2023) and inhibitory effects (Blomeley and Bracci, 2005) on excitatory transmission through ACh neurons.

Recently, Belilos et al. demonstrated that substance P, which causes lasting excitation of ChIs (Francis et al., 2019), is responsible for rebound ACh release during aversive foot shock. In fact, the amplitude of the rebound scaled with the conditioned freezing response elicited by cue/foot shock pairings. However, the foot shock, not the conditioning itself, produced the release. Therefore, substance P could be partially responsible for this response when an animal encounters a salient, aversive stimulus. It is also possible that substance P, which is released in response to rewarding stimuli (Commons, 2010; Vanderah and Sandweiss, 2015), could also be accountable for the rebound response in these reward-related conditions as well. However, this has yet to be demonstrated. In fact, activating D1-MSNs at higher frequency stimulation produces both a pause through the inhibition of ChIs via GABA_A_ and GABA_B_ receptors followed by excitation via the NK1 receptor (Francis et al., 2019). It remains to be seen if other excitatory peptides or disinhibition mechanisms may also provide a source of excitation during the rebound. Other neuromodulators and peptides that could participate in the response are discussed below.

## 4 Other neurotransmitters and neuromodulators controlling cholinergic activity

ChIs express many other neurotransmitter, neuromodulator, and neuropeptide receptors, and their activity elevates or suppresses tonic spontaneous firing rates, fine-tuning striatal circuitry. In some cases, the functional effect on activity is not fully understood. However, unlike most other neuromodulators, peptides provide a broad signal and can signal through volume transmission, which can spatially synchronize the activity of ChIs and other neurons. While the role of these neuromodulators in the conditioned pause response is not known, these factors may be shown to be involved in phasic ChI responses in the future.

### 4.1 Serotonin

The raphe nuclei send dense serotonergic projections to the striatum (Steinbusch, 1981; Lavoie and Parent, 1990). ChIs express several serotonin [5-HydroxyTryptamine (5-HT)] receptor subtypes, including 5-HT_1_, 5-HT_2C_, 5-HT_6_, and 5HT_7_, which modulate potassium ion currents and enhance neuronal excitability (Rada et al., 1993; Blomeley and Bracci, 2005; Bonsi et al., 2007). Immunolabeling studies have detected greater expression of 5-HT_1A_ and 5-HT_1B_ on ChIs of the ventral striatum, with little to no detection of these receptors in the DSt. Conversely, 5-HT_7_ receptors are more abundantly expressed in the DSt, while 5-HT_5A_ receptors show equal expression in both regions (Steinbusch, 1981; Virk et al., 2016). In rats, *in vivo* infusion of 5-HT in NAc results in decreased ACh release, which was blocked by 5-HT_1_ antagonist (Rada et al., 1993). Upon serotonin (5-HT) application, ChIs in the DSt are depolarized, whereas those in the ventral striatum show hyperpolarization (Virk et al., 2016). Serotonin-mediated ChI depolarization may be due to 5-HT_2_ receptors as bath application of selective agonist (α-methyl-5-HT) for 5-HT_2_ receptor significantly increased spontaneous firing in ChIs and is blocked by 5-HT_2_ antagonist (Blomeley and Bracci, 2005). Rapid serotonin release may provide another signal to directly depolarize or hyperpolarize ChIs during phasic responses, albeit in a region-selective and concentration-dependent manner.

### 4.2 Opioids

ChIs express the mu-opioid receptor (MOR) and delta-opioid receptor (DOR), members of the GPCR family. Activation of these results in decreased spontaneous firing, resulting in reduced ACh release (Mulder et al., 1984; Svingos et al., 2001; Jabourian et al., 2005; Pérez et al., 2007; Arttamangkul et al., 2021). The double immunostaining experiment indicates MOR expression is restricted to ChIs in limbic/prefrontal territory but not in the sensorimotor area of the DSt. MOR is also reported to have diurnal variation in its expression, which is higher in the afternoon (last phase of light period) than in the morning (start of light period) in rats (Jabourian et al., 2005), which corresponds to the sleep phase of the rodents. *In situ* hybridization of DOR mRNA is seen in ChIs in the striatum (Le Moine et al., 1994; Svingos et al., 1998; Heath et al., 2018). Substance P induces delta receptor accumulation in the somatic area of ChIs in the NAc shell via the activation of NK1R (Heath et al., 2018). In addition, a GFP-tagged DOR knock-in mouse showed functionally expressed DOR, indicating its postsynaptic localization on ChIs (Le Moine et al., 1994; Scherrer et al., 2006). The inhibition observed in the current–voltage relationship for MOR suggests that inward rectification mediated by the GIRK channel drives inhibition (Britt and McGehee, 2008). Receptor desensitization occurs following simultaneous activation of MOR and DOR, but agonist-induced internalization is observed only for DOR (Faget et al., 2012; Arttamangkul et al., 2021). This finding indicates that MOR and DOR receptors may function independently of one another and may be activated in certain conditions. In addition, dynorphin, which is released from D1-MSNs and acts on ChIs via the kappa opioid receptor (KOR) can excite ChIs at low concentrations (Crain and Shen, 1996), and high concentrations can inhibit ChIs (Gross et al., 1990). Overall, activation of these receptors within the striatum may act as a brake on ChI activity, especially when released at higher levels. However, rapid, phasic changes in ChI activity by opioids is still not well understood.

### 4.3 Corticotrophin releasing factor

CRF is released into NAc in response to salient environmental stimuli, such as stress and novel objects (Lemos et al., 2012; Holly et al., 2016; Williams et al., 2022). CRF acts via CRF receptor type-1 (CRF-R1) and type-2 (CRF-R2), which are broadly expressed across the brain (Van Pett et al., 2000). However, RNAscope *in situ* hybridization has shown that *Crh 1* mRNA is expressed in ChAT-positive neurons, while *Crh 2* is absent in these regions (Van Pett et al., 2000; Lemos et al., 2019). CRF is expressed in a heterogenous population of MSNs in the ventral portion of the striatum (Lemos et al., 2019) and can be released by collaterals (Eckenwiler et al., 2024). Notably, CRF mRNA expression is high in the NAc but lacking in the DSt (Lemos et al., 2019), suggesting that regional differences in the expression may contribute to behavioral regulation. Activation of CRF receptors leads to cAMP-dependent activation of ChIs, elevating ACh levels as demonstrated through *in vivo* microdialysis in the NAc (Chen et al., 2012). Bath application of CRF enhances the spontaneous firing of ChIs across different regions and has been observed to robustly increase ChI firing in the NAc shell (Lemos et al., 2019; Ingebretson et al., 2024) and DSt (Lemos et al., 2019). Therefore, CRF may be able to alter several components of the phasic response, including DA release, or provide direct excitation of ChIs like substance P.

### 4.4 Galanin, leptin, and insulin

Galanin, Leptin, and Insulin are peptides that interact and regulate several processes related to feeding (Morton et al., 2006; Wu et al., 2012; Myers and Olson, 2014; Le et al., 2020; Affinati et al., 2023; Dornbush and Aeddula, 2024). Galanin, a 29 (mouse) or 30 (human) amino acid peptide primarily co-released mainly from neuronal populations in the locus coeruleus region along with other regions, has been linked to stress regulation, and conditions like depression, anxiety, and substance abuse (Weiss et al., 2006; Kuteeva et al., 2008; Juhasz et al., 2014; Hökfelt et al., 2018; Genders et al., 2020). Its action is mediated through galanin receptors (GALRs) divided into three subtypes GALR_1_, GALR_2_, and GALR_3_ (Wang and Gustafson, 1998; Branchek et al., 2000). *In situ* hybridization studies have shown that GALR_1_ mRNA is expressed in the striatum, with a distribution pattern resembling that of ChIs in the NAc (Zachariou, 2000). Galanin can stimulate striatal ACh release via GALRs on ChIs, which could be mediated by GALR_1_ (Ogren and Pramanik, 1991; Pramanik and Ogren, 1993; Zachariou, 2000). This effect depends on the anesthesia state of the animal, as awake-behaving rodents displayed decreased ACh release (Antoniou et al., 1997).

Leptin, a 146 amino acid peptide hormone released from adipose tissue, activates leptin receptors (LepR), releasing DA via nAChR (β2 containing) mechanisms (Wada et al., 2014; Mancini et al., 2022), by increasing ChI excitability. ChIs also express insulin receptors on ChIs (Stouffer et al., 2015), and, unlike Leptin, insulin, a 51 amino acids peptide, is reported to be expressed by some neuronal populations in brain, but their projections to the striatum are not known (Eerola et al., 2022; Dakic et al., 2023). Regardless of the source, peripheral or central, insulin enhances ChI excitability in all striatal subcompartments (Patel et al., 2023). This enhanced activity can also cause DA release via nAChR signaling. Therefore, these peptides may be a candidate for activity during rebound, particularly in the context of feeding and reward.

### 4.5 Estrogen

Estrogen exerts its effects in the CNS primarily via receptors ERα and ERβ (Eyster, 2016). Sex-dependent activity patterns have been observed in ChIs in the NAc (Ingebretson et al., 2024), suggesting ChI tonic activity may be regulated by estrogen signaling. However, direct application of 17β-estradiol has minimal to no effect on ChI spontaneous firing activity (Kövesdi et al., 2023). Using RNAscope in situ hybridization, estrogen receptors have been detected in subpopulations of ChIs in the striatum (Kövesdi et al., 2023). Ultrastructural analysis further indicates that ERα is expressed on ChIs at lower levels (Almey et al., 2012). Although estrogen receptors are expressed on ChIs, estrogen interacts with the striatal dopaminergic system, indirectly influencing ACh’s local release (Euvrard et al., 1979).

## 5 Outcomes of altering ChI activity and ACh release

Temporal signaling allows for synchronous and broad cholinergic signaling through large areas of the striatum, which controls the activity of MSNs or facilitates short-term or long-term plasticity in MSNs, thus controlling responses to salient environmental stimuli. Temporally selective, acute release of ACh alters intrinsic excitability. During a synchronous ChI pause, less ACh is released leading to diminished post-synaptic M1 receptor signaling and MSNs undergo less additional depolarization in the upstate (Zucca et al., 2018). This depolarization suppression effectively diminishes activity of MSNs when the membrane potential is near the action potential threshold. *In vivo,* it has been shown that activation of ChIs disrupts spiking of MSNs by causing a suppression in activity (Crittenden et al., 2017), potentially through the same mechanism. Oppositely, repeated activation of ChIs can lead to a lasting, M1 receptor-dependent increase in MSN excitability (Lv et al., 2017), suggesting a bidirectional control of MSN excitability by M1 receptor signaling putatively through suppression or disinhibition of Kv4 potassium channel currents (Nakamura et al., 1997) or N- and L-type calcium channels (Perez-Burgos et al., 2008; Howe and Surmeier, 1995). Overall, these results indicate that M1 receptor activation by suppression or enhancement of ACh release can oppositely control intrinsic excitability of MSNs.

ACh has profound effects on excitation within the striatum and altering ChI activity phasically influences the outcome of excitatory input via dendritic excitability and long-term plasticity including LTP and LTD of excitatory transmission which is dependent on receptor subtype activation (Oldenburg and Ding, 2011). Phasic activity of ChIs can activate pre-synaptic M2 and M4 receptors which are expressed on glutamatergic afferents, suppressing glutamate release from cortical terminals on MSNs (Pancani et al., 2014; Hersch et al., 1994; Barral et al., 1999; Pakhotin and Bracci, 2007; Ding et al., 2010). Aligned with these findings, M2 receptor inhibition can enhance the expression of LTP on MSNs (Calabresi et al., 1998b). Other pre-synaptic ACh receptors can modulate glutamate release as well. Nicotinic receptor activation facilitates or causes glutamate release onto MSNs depending on the subunit (Howe et al., 2016; Assous, 2021). It remains unclear how M2, M4, and nicotinic receptor activation temporally locks glutamate release on MSNs to the phasic burst-pause-rebound response. However, it is known that nicotinic receptors can promote the release of glutamate on various GABAergic interneuron populations and therefore provide inhibition of MSNs. Pause excitation sequences lead to inhibition of MSNs *in vivo* and *ex vivo* via nAChR-mediated activation of NPY- Nerve growth factor (NGF) GABA neurons (English et al., 2012; Faust et al., 2016). Further, inhibition of ACh release during a pause suppresses cortical glutamate input. This excitatory suppression is due to a decrease in post-synaptic M1 receptor signaling which decreases the dendritic excitability of MSNs and suppress the size of excitatory responses (Wang et al., 2006). An increase in M1 receptor signaling, however, enhances dendritic excitability by suppressing Kir2 dendritic inhibition selectively in D2-MSNs (Shen et al., 2007) and may play a role in enhancing excitation to D2-MSNs during the thalamic-mediated burst-pause response (Ding et al., 2010). These outcomes provide M1, M2, M4, and nicotinic receptors with the ability to acutely modulate excitation during phasic bursts and pauses. It is still unclear how pre-synaptic and post-synaptic mechanisms coordinate glutamatergic transmission to provide an overall increase or decrease in glutamatergic signaling. Based on the current literature, we predict that post-synaptic signaling may play a larger role in long-term synaptic plasticity.

In line with changes in dendritic excitability, suppression of M1 receptor signaling restores endocannabinoid LTD induction even in the absence of D2 receptor signaling, which is critically involved in corticostriatal LTD (Wang et al., 2006). Induction of LTP in conditions where NMDA receptors are active at resting states (i.e., low magnesium conditions), requires M1 receptor signaling (Calabresi et al., 1999). Often, ACh release inversely covaries with DA release to promote plasticity on MSN subtypes. A coordinated decrease in ACh release in the DSt during the pause with matched DA release is sufficient to induce sensory-evoked LTP on MSNs *in vivo* (Reynolds et al., 2022). When ACh and DA release are mismatched, that is when ACh and DA are both high, MSNs can display sensory-evoked LTD. However, the mismatch may provide subregion-selective information and could vary across these regions (Duhne et al., 2024). Cell-type activation of mAChRs selects the expression of plasticity. In D1-MSNs, M4 receptor activation promotes excitatory LTD on D1-MSNs and blocks LTP (Shen et al., 2015). Pauses in DA during the putatively high activity of ACh have been shown to enhance spine formation and LTP during discrimination learning selectively on NAc D2-MSNs (Schulz and Reynolds, 2013; Iino et al., 2020), likely through muscarinic receptor activation (Calabresi et al., 1998b; Francis et al., 2019; Poppi et al., 2021). In addition, while the pause itself is important for inducing plasticity, the rebound facilitates plasticity in the NAc in response to aversive stimuli. The rebound correlates with aversive learning in mice, is substance P dependent, and facilitates LTP selectively on D2-MSNs (Belilos et al., 2023). These DA pauses and ACh mediated D2-MSN plasticity may be overlapping mechanisms to facilitate excitatory LTP on D2-MSNs. In contrast, pauses or transient decreases in ACh release can allow for corticostriatal LTP on D1-MSNs (Laverne et al., 2022). This suggests these responses could aid in facilitating cell-type selective excitatory plasticity on the major projection neurons of the striatum. Due to the multi-factor requirement of each phase, eliminating one molecule that participates in this response can have drastic effects on the response. This would provide precise, input-specific, cell-type specific, and striatal sub-region selective control over ACh-dependent plasticity in the striatum.

## 6 Future directions

The conditioned pause response is one of the key mechanisms ChIs can use to facilitate changes in MSN activity and plasticity. We summarize how glutamatergic inputs from cortical and thalamic inputs can facilitate an initial burst followed by rapid release of DA and GABA, mediating a pause response and a rebound mediated by intrinsic properties and peptide release. The mechanisms of the burst and pause are quite clear and very well studied. However, the rebound and what drives the extended activation of the rebound is much less well studied. Information about the temporal release of molecules, particularly across striatal sub-compartments, would provide a better understanding of how selective plasticity drives behavioral outcomes in response to phasic changes in ChI activity.

Along with collecting information about mechanisms controlling each phase of the conditioned pause response, we have discussed one peptide, substance P, which participates in mediating the rebound response (Belilos et al., 2023). Neuropeptides are known to modulate various aspects of the neuronal function, often in a context and time-dependent manner, suggesting that they could critically shape the response of ChIs to salient stimuli. The peptides we review here excite ChIs and can facilitate the release of DA. We suggest other neurotransmitters, neuromodulators, and neuropeptides may facilitate the long rebound observed following the pause, potentially in a context-dependent manner. This would require additional studies to assess how other peptides may play a temporal-dependent role in the phasic excitation of ChIs. These studies should assess neuropeptide release in coordination with classical neurotransmitter release on ChIs, including glutamate, which facilitates the release of these peptides by strongly stimulating local striatal neurons. Furthermore, understanding how other peptides that activate ChIs may be involved in the rebound response would provide a better understanding of how stimuli with different valences or contexts may enhance some of these responses over others.

Although few, striatal ChIs are critical for the striatal output, guiding short-term and long-term changes in projection neuron output. Yet, refined control of ChI activity remains partially understood. The studies described here indicate that altering the ChI firing rate is multifaceted, requiring a variety of intrinsic ChI properties and extrinsic neurotransmitters and neuromodulators. Furthermore, striatal subregion-specific differences and cell-type-specific differences are observed. Understanding the control of ChI activity provides a foundation for understanding how the striatum engages in associative responses and learning underlying learning, reward, and aversion.
